# Examining the relationship between health-related need and the receipt of care by participants experiencing homelessness and mental illness

**DOI:** 10.1186/1472-6963-14-404

**Published:** 2014-09-18

**Authors:** Lauren B Currie, Michelle L Patterson, Akm Moniruzzaman, Lawrence C McCandless, Julian M Somers

**Affiliations:** Faculty of Health Sciences, Simon Fraser University, 8888 University Drive, Burnaby, V5A 1S6 BC Canada

**Keywords:** Homelessness, Health services, Unmet need, Mental illness

## Abstract

**Background:**

People experiencing homelessness and mental illness face multiple barriers to care. The goal of this study was to examine the association between health service use and indicators of need among individuals experiencing homelessness and mental illness in Vancouver, Canada. We hypothesized that those with more severe mental illness would access greater levels of primary and specialist health services than those with less severe mental illness.

**Methods:**

Participants met criteria for homelessness and current mental disorder using standardized criteria (n = 497). Interviews assessed current health status and involvement with a variety of health services including specialist, general practice, and emergency services. The 80^th^ percentile was used to differentiate ‘low health service use’ and ‘high health service use’. Using multivariate logistic regression analysis, we analyzed associations between predisposing, enabling and need-related factors with levels of primary and specialist health service use.

**Results:**

Twenty-one percent of participants had high primary care use, and 12% had high use of specialist services. Factors significantly (p ≤ 0.05) associated with high primary care use were: multiple physical illnesses [AOR 2.74 (1.12, 6.70]; poor general health [AOR 1.68 (1.01, 2.81)]; having a regular family physician [AOR 2.27 (1.27, 4.07)]; and negative social relationships [AOR 1.74 (1.01, 2.99)]. Conversely, having a more severe mental disorder (e.g. psychotic disorder) was significantly associated with lower odds of high service use [AOR 0.59 (0.35, 0.97)]. For specialist care, recent history of psychiatric hospitalization [AOR 2.53 (1.35, 4.75)] and major depressive episode [AOR 1.98 (1.11, 3.56)] were associated with high use, while having a blood borne infectious disease (i.e., HIV, HCV, HBV) was associated with lower odds of high service use.

**Conclusions:**

Contrary to our hypotheses, we found that individuals with greater assessed need, including more severe mental disorders, and blood-borne infectious diseases had significantly lower odds of being high health service users than those with lower assessed needs. Our findings reveal an important gap between levels of need and service involvement for individuals who are both homeless and mentally ill and have implications for health service reform in relation to the unmet and complex needs of a marginalized sub-population. (Trial registration: ISRCTN57595077 and ISRCTN66721740).

## Background

In Canada and throughout the developed world, homelessness is a significant social issue that demands the attention of our public institutions. A staggering proportion of those experiencing homelessness are also experiencing mental disorders, demanding high levels of health care service to meet the needs of these individuals [1, 2]. Previous research has concluded that inadequate services are available for people experiencing homelessness and mental illness, often due to competing priorities, barriers to treatment access, and poor discharge planning and follow-up [3, 4]. However, little is known about the association between varying complexities of need (e.g., type of mental disorder, multiple mental disorders, co-morbid conditions, substance use, criminal justice system involvement) and levels of health service use.

Individuals experiencing homelessness and mental illness are a heterogeneous population requiring varying levels of health and social supports. Discontinuity between services for people with complex needs (e.g., concurrent disorders), poor psychiatric follow-up, an absence of low-barrier treatment options, stigma, and discrimination each contribute to high levels of unmet need within this population [5–7]. Previous research has shown that homeless individuals underuse outpatient services and, as a result, rely heavily on emergency department visits and inpatient stays to address both physical and mental illnesses [3, 8, 9]. In response, researchers and service providers have called for the reorientation of health and social services to a more individualized and client-centered approach [3, 4, 10, 11]. A challenge in advocating for such service reorientation is the lack of empirical research describing the distinct needs of subgroups within the homeless mentally ill population [12]. In order to orient services in a manner that best addresses the needs of different individuals, it is important to identify the factors associated with different levels of health service use and unmet need.

A challenge to understanding discontinuities in health service use is identifying the unique and diverse needs of this population and matching individuals with differing levels of care. The *Gelberg-Andersen Behavioural Model for Vulnerable Populations* offers a framework to help identify factors associated with health service use with the aim of improving healthcare access and delivery [13–15]. Previous research using this model has shown that, among homeless individuals, there are specific characteristics that can help to predict and explain service involvement, and are categorized as *predisposing, enabling,* and *need-related* factors*.* Predisposing factors include individual characteristics, (e.g., age, gender ethnicity, education, history of homelessness), and are associated with commonly observed demographic trends in health seeking behaviour. Enabling factors are comprised of systemic and structural considerations such as having a regular family physician, social support, or access to health care, and exert an influence via the availability and accessibility of health care services. Finally, need-related factors consist of perceived and objective medical need and include mental and physical health status, severity and type of illness, and substance use [13, 14, 16].

However, this model has not been applied to a sample of homeless individuals wherein all participants also have a mental disorder, with or without a concurrent substance use disorder [13–15]. Furthermore, previous applications of the Gelberg-Andersen model have primarily been in the context of the American healthcare system, where structural aspects of funding have an important bearing on access to healthcare.

Existing research suggests that individuals experiencing more complex mental disorders, such as psychotic disorders, require a higher level of service compared to individuals with less severe mental disorders [15, 17]. It is therefore hypothesized that individuals with more complex needs, including those experiencing more severe mental disorders, multiple comorbidities and concurrent disorders will have a greater number of encounters with both primary and specialist health care than individuals with less complex needs.

By examining factors shown to be associated with different levels of service use, we can help to identify gaps in the current service landscape, and target services to address areas of unmet need. Guided by the Gelberg-Andersen model, the purpose of this research is to examine the association between level of health service use with predisposing, enabling, and need-related factors among a sample of participants experiencing homelessness and mental illness in Vancouver, Canada. The empirically derived Gelberg-Andersen model will be used as a framework for this analysis with the goal of identifying potential discontinuities in care and opportunities for intervention.

## Methods

### Data source and sample

Data were drawn from baseline interviews for the full sample (n = 497) of participants enrolled in the *Vancouver At Home* (VAH) study. Participants recruited to the VAH study met inclusion criteria for recent homelessness and current mental illness as assessed through the use of standardized assessment measures administered in person by trained interviewers [18]. Participants were recruited from over 40 different community and institutional agencies, representing roughly 13 different types of services [18]. Referral sources included homeless shelters, drop-in centres, homeless outreach teams, hospitals, community mental health teams, and criminal justice programs. Prospective participants were contacted directly by research team members or were referred to the VAH research team by agency staff. Final eligibility was confirmed with an in-person screening interview. Approximately 800 individuals were assessed for eligibility. Among those, roughly 300 were excluded due to: ineligibility (n ~ 200); being eligible, but losing contact following screening (n = 100); declining to participate (n = 3); and not being able to complete the baseline interview (n = 3) [18]. All participants were at least 19 years of age and provided written, informed consent prior to participating in the study.

VAH is a longitudinal study, consisting of two randomized control trials (RCTs) investigating housing and supports for people experiencing homelessness and mental illness [18]. With the RCT design participants were randomly assigned to one of 5 different study arms each consisting of approximately 100 participants. Sample size calculations were performed prior to recruitment to ensure sufficient power to perform outcome analysis between groups. Sample sizes of 100 participants per arm were determined based on effect size estimates of 0.5 for major outcome variables, power of 0.80 (β = 0.20) [18, 19]. Analyses presented in the current study consider only baseline data from the full sample of VAH participants prior to randomization. The study is part of a Canadian multi-centre project which took place from October 2009 – March 2013 [19].

### Predisposing, enabling and need factors

Data concerning socio-demographic characteristics, health service use, housing histories, mental illness, substance use and quality of life were collected through a series of self-report questionnaires and categorized into the domains of predisposing, enabling or need-related factors. The selection of explanatory variables and categorization into the three different domains followed the procedures of previous investigators [13, 16] and the guidelines for implementing the Andersen-Newman and Gelberg-Andersen models [13–15].

#### Predisposing factors

Predisposing factors included sociodemographic characteristics as follows: gender (male/female), age [Youth (<25); 25–44; and > 44], education (incomplete high school; graduated high school), marital status (single/never married; married/partnered; separated/widowed/divorced), and whether they had a child 18 years or younger (yes/no). Self-reported ethnicity was categorized as: Caucasian, Aboriginal and Other. Housing status was assessed based on shelter use in the past 6 months (yes/no), lifetime duration of homelessness (1–3 years; >3 years); longest single period of homelessness (1 year; >1 year), and current housing status (absolutely homeless versus precariously housed) (See Goering et al. [19]). Criminal justice involvement was assessed in terms of having been in jail in the past 6 months (yes/no).

#### Enabling factors

Personal and social resources were categorized as enabling factors including: having a regular family physician (yes/no) and having a place to go to seek health care (yes/no). Unmet need was assessed by asking participants if, in the past year, they felt they needed health care but did not receive it (yes/no). Social resources were assessed in terms of the type and quality of social relationships, including general feelings about family, types of daily activities, the amount of time spent with other people, and the people they interact with socially (Quality of Life Interview-20 [20]).

#### Need factors

Need related factors included variables concerning physical and mental health. Physical health was assessed through self-reported physical illness including: blood-borne infectious diseases (HIV, Hepatitis C and/or Hepatitis B), chronic illnesses (heart disease, cancer, COPD, etc.) history of head injury (yes/no), and having multiple physical illnesses (≥2). General health was evaluated on a five-point Likert scale ranging from excellent to poor. Responses were dichotomized as positive (excellent/very good/good) or negative (fair/poor) perceived health. Mental disorders, substance dependence and alcohol dependence were assessed using the MINI International Neuropsychiatric Interview [21]. Mental disorders were dichotomized into clusters of less severe form (major depressive episode, panic disorder, post-traumatic stress disorder) and severe form (mood disorder with psychotic features, psychotic disorder, and manic or hypomanic episode). Multiple mental disorders were assessed as meeting criteria for two or more (≥2) disorders.

### Definition of high and low health service use

Service use was evaluated based on the frequency of past-month primary health care (family doctor, nurse, dentist, or pharmacist) or specialist health care (specialist physician, psychologist, psychiatrist, addiction worker or mental health worker) visits. The 80^th^ percentile was used to define two groups whereby two or fewer visits (<3) for each type of service in the past month were categorized as ‘low health service use’ and three or more visits (≥3) were categorized ‘high health service use’.

### Statistical analysis

Pearson’s Chi-square tests were used to conduct pair-wise comparisons between predisposing, enabling and need-related baseline characteristics, among low and high service use groups for both primary and specialist health care providers. Bivariate and multivariate logistic regression analyses were used to estimate baseline associations between various predisposing, enabling and need-related factors and levels of primary and specialist health care. Variables were selected using the Gelberg-Andersen framework for the regression analysis. We used a significance level of p ≤ 0.10 to select variable for inclusion in the multivariable logistic regression analyses. Stepwise logistic regression (backwards elimination) was used to select variables for the final multivariable model. Odds ratios and 95% confidence intervals obtained through logistic regression were reported as effect sizes. All reported p-values were 2-sided. SPSS v21 software was used to conduct all statistical analyses. Institutional review and ethics approval was provided by Simon Fraser University’s Office of Research Ethics, under the application entitled “Research Demonstration Project on Housing and Mental Health in Vancouver, BC”, application number 2009 s0231.

## Results

### Sample characteristics

The median age of participants (n = 497) was 41 years, and the majority were male (73%), born in Canada (87%), of European (57%) or Aboriginal (15%) decent, and met criteria for absolute homelessness (78%). The median duration of lifetime homelessness was 36 months and the median age of first homelessness was 28 years. Most participants were single and never married (70%), unemployed (96%), and 41% had not completed high school [18].

The most prevalent mental disorders in the sample were psychotic disorder (53%) and major depressive episode (40%), followed by post-traumatic stress disorder (PTSD) (26%), panic disorder (21%) and (hypo) manic episode (19%). Half (52%) of participants met criteria for two or more mental disorders. Substance dependence was observed among 58% of participants and alcohol dependence among 24%, with 28% of the sample reporting poly-drug use (two or more types) and 29% reporting daily illicit drug use [22]. Physical illnesses, including infectious and chronic conditions, were highly prevalent, with most participants (81%) reporting having two or more physical illnesses including the presence of hepatitis C among 30% of participants [18].

In the month prior to recruitment, 49% of participants reported being seen by a health service provider and 27% by a psychiatrist. Historically, 53% of participants had been hospitalized for a mental illness two or more times in the preceding five-years, and 12% had been hospitalized for more than 6 months in the same time period. In the preceding 6 months, the majority of participants (58%) had visited an emergency room and 40% had arrived at a hospital via ambulance.

### Health service use – past month

In order to examine the nature of health service use among participants, visits were categorized as primary care or specialist care visits. For primary care, 393 (79%) participants were categorized as low use (<3 visits) and 103 (21%) as high use (≥3 visits). For specialist care, 437 (88%) were categorized as low use (<3 visits) and 60 (12%) as high use (≥3 visits).

Univariate associations between the outcome (levels of service use) and predictor variables are presented in Tables 1, 2 and 3, sorted by primary and specialist health service use. Within the primary health service use category, none of the observed associations between predisposing factors and levels of service use were significant at the p < 0.05 level; while the only predisposing variables significant at the p ≤ 0.10 level were ethnicity, marital status and having children under 18 years. Within the specialist health service use category, age at enrolment and being ‘hospitalized two or more times for a mental illness in the past 5 years’ were significantly associated with level of specialist health service use (p < 0.05). These variables as well as education level and duration of longest single period of homelessness, were included in multivariable regression analyses.

**Table 1:** **Univariate comparisons of predisposing characteristics, by primary and specialist health service use**

Variable		Primary health service use			Specialist health service use		
	All N (%)	Low use (<3 visits) N (%)	High use (≥3 visits) N (%)	P value	Low use (<3 visits) N (%)	High use (≥3 visits) N (%)	P value
Male gender	358 (73)	288 (74)	70 (69)	0.292	316 (73)	43 (72)	0.830
Age at enrolment visit							
Youth	36 (7)	28 (7)	8 (8)	0.889	27 (6)	9 (15)	**0.031**
25-44 years	280 (57)	224 (57)	56 (54)		253 (58)	28 (47)	
> 44 years	180 (36)	141 (36)	31 (38)		157 (36)	23 (38)	
Ethnicity							
Aboriginal	77 (15)	61 (16)	16 (16)	**0.068**	71 (16)	6 (10)	0.433
Caucasian	279 (56)	212 (54)	67 (65)		245 (56)	35 (58)	
Other	140 (28)	120 (31)	20 (19)		121 (28)	19 (32)	
Education (≤Grade 8)	76 (15)	62 (16)	14 (14)	0.840	65 (15)	11 (18)	**0.093**
Single marital status	342 (70)	278 (72)	64 (62)	**0.067**	301 (70)	42 (70)	0.939
Have children (under 18)	122 (25)	89 (23)	33 (32)	**0.059**	108 (25)	14 (25)	0.920
Hospitalized for mental illness (>6 months) in past 5 years	57 (12)	49 (13)	8 (8)	0.164	49 (11)	8 (13)	0.666
Hospitalized for mental illness (>2 times) in past 5 years	253 (53)	206 (54)	47 (47)	0.190	213 (50)	40 (71)	**0.003**
Worked continuously at least one year in the past	322 (65)	257 (66)	65 (63)	0.597	280 (65)	43 (72)	0.275
Jail in last 6 months	68 (14)	53 (14)	15 (15)	0.777	06 (14)	8 (13)	0.933
Shelter in last 6 months	143 (29)	113 (29)	30 (29)	0.941	127 (29)	16 (27)	0.701
Duration of homelessness in lifetime							
1-3 Years	256 (52)	208 (54)	48 (47)	0.197	223 (52)	34 (57)	0.474
3 Years Plus	234 (48)	179 (46)	55 (53)		208 (48)	26 (43)	
Duration of homelessness -longest single period							
1 Year	246 (50)	190 (49)	56 (54)	0.330	210 (49)	36 (60)	0.102
1 Year Plus	245 (50)	198 (51)	47 (46)		221 (51)	24 (40)	
Age of first homelessness (<25 years)	214 (44)	166 (43)	48 (47)	0.427	191 (44)	23 (38)	0.381
Housing Status (Absolutely Homeless)	388 (78)	313 (80)	75 (73)	0.135	342 (78)	23 (38)	0.780

Bolded p-values indicate significance at p ≤ 0.10.

**Table 2:** **Univariate comparisons of enabling characteristics, by primary and specialist health service use**

Variable		Primary health service use			Specialist health service use		
	All N (%)	Low use (<3 visits) N (%)	High use (≥3 visits) N (%)	P value	Low use (<3 visits) N (%)	High use (≥3 visits) N (%)	P value
Regular Family Physician	320 (65)	241 (61)	79 (78)	**0.002**	277 (64)	43 (72)	0.217
Regular place to go for health care	394 (81)	304 (79)	90 (88)	**0.031**	341 (80)	54 (90)	**0.053**
Needed health care but didn’t receive it (past year)	209 (43)	155 (41)	54 (53)	**0.026**	189 (44)	20 (35)	0.154
Feelings about family in general	199 (43)	147 (41)	52 (54)	**0.013**	176 (43)	23 (40)	0.595
Feelings about things you do with other people	117 (25)	80 (21)	37 (37)	**0.001**	101 (24)	16 (27)	0.607
Feelings about amount of time spent with other people	151 (31)	116 (30)	35 (35)	0.341	138 (33)	13 (22)	0.119
Feelings about people seen socially	136 (28)	104 (27)	32 (32)	0.337	120 (28)	16 (27)	0.792

Bolded p-values indicate significance at p ≤ 0.10.

**Table 3:** **Univariate comparisons of need-related characteristics, by primary and specialist health service use**

Variable		Primary health service use			Specialist health service use		
	All N (%)	Low use (<3 visits) N (%)	High use (≥3 visits) N (%)	P value	Low use (<3 visits) N (%)	High use (≥3 visits) N (%)	P value
Major Depressive Episode	199 (40)	147 (37)	52 (51)	**0.016**	168 (38)	31 (52)	**0.050**
Manic or Hypomanic Episode	97 (20)	80 (20)	17 (17)	0.380	84 (19)	13 (22)	0.654
Post Traumatic Stress Disorder (PTSD)	129 (26)	93 (24)	36 (35)	**0.021**	113 (26)	16 (27)	0.901
Panic Disorder	104 (21)	80 (20)	24 (23)	0.513	91 (21)	13 (22)	0.880
Mood Disorder with Psychotic Features	84 (17)	68 (17)	16 (16)	0.698	73 (17)	11 (18)	0.758
Psychotic Disorder	263 (53)	218 (56)	44 (43)	**0.021**	236 (54)	27 (45)	0.190
Suicidality (moderate/high)	168 (34)	128 (33)	40 (39)	0.232	144 (33)	24 (40)	0.234
Multiple mental disorders (≥2)	240 (48)	179 (46)	61 (59)	**0.013**	207 (47)	33 (55)	0.267
Less severe cluster of mental disorder	264 (53)	194 (49)	70 (68)	**0.001**	230 (53)	34 (57)	0.557
Severe cluster of mental disorder	363 (73)	299 (76)	63 (61)	**0.002**	318 (73)	45 (75)	0.715
Alcohol dependence	121 (24)	95 (24)	26 (25)	0.822	104 (24)	17 (28)	0.443
Substance dependence	288 (58)	217 (55)	71 (69)	**0.012**	257 (59)	31 (52)	0.293
Any physical illness	453 (91)	355 (90)	98 (95)	0.122	398 (91	55 (92)	0.880
Blood-borne Infectious diseases (HIV/HCV/HBV)	157 (32)	113 (29)	44 (43)	**0.009**	145 (34)	12 (20)	**0.042**
Multiple physical illness (≥2)	402 (81)	306 (78)	96 (93)	**0.000**	353 (81)	49 (82)	0.870
Head injury	270 (56)	211 (56)	58 (57)	0.830	234 (55)	36 (62)	0.322
General Health (fair/poor)	235 (48)	171 (44)	64 (62)	**0.001**	211 (48	24 (40)	0.222

Bolded p-values indicate significance at p ≤ 0.10.

Table 2 presents the results of chi-square tests for enabling factors. All variables pertaining to health care access were significantly associated with past month health service use in the primary care category (p < 0.05), and were included in the regression model. In the specialist care category, only ‘having a regular place to go for health care’ was significant at the p < 0.05 level. Measures related to quality of life were assessed for inclusion in the regression models. For primary care, both ‘feelings about family in general’ and ‘feelings about the things done with other people’ were significantly associated with levels of service use and thus included in the regression model (p < 0.05). In the specialist care category, none of the variables were significantly associated with level of service use and only ‘feeling about the amount of time spent with other people’ was selected for inclusion in the regression model (p ≤ 0.10).

Several need-related factors were significantly associated with levels of service use (see Table 3). In the specialist health service use category, only major depressive episode and blood-borne infectious disease were significantly associated with level of service use at the p < 0.05 level and no additional variables were included at the p ≤ 0.10 level.

Tables 4 and 5 present the results of univariate and multi-variable logistic regression analyses. Unadjusted odds ratios are included for all variables that met the threshold for inclusion in the logistic regression analysis (p ≤ 0.10). For primary health service use (Table 4), having two or more physical illnesses, reporting poor general health, having a regular family physician, and feeling ‘horrible’ about the ‘things that they do with others’ were all significantly associated with high primary health service use. By contrast, participants with more severe mental disorders were significantly less likely to have high primary health service use than those without severe mental disorders. Ethnicity, having a regular location for seeking health services, self-assessed unmet health care need, current substance dependence, and blood-borne infectious diseases were not significantly associated with level of health service use in the final regression model.

**Table 4:** **Associations between predictor variables and high primary health service use (≥3 visits)**

Outcome variable	Unadjusted OR (95% CI)	P value	Adjusted OR (95% CI)*	P value
**Predisposing Factors**				
Ethnicity				
Aboriginals	1.57 (0.76, 3.25)	0.221		
Caucasian	1.90 (1.10, 3.28)	**0.022**		
Other				
Single marital status	1.53 (0.97, 2.41)	0.069		
Have children (under 18)	1.58 (0.98, 2.55)	0.061		
**Enabling Factors**				
Regular Family Physician	2.17 (1.31, 3.60)	**0.003**	2.27 (1.27, 4.07)	**0.006**
Regular place to go for health care	2.02 (1.06, 3.88)	**0.034**		
Needed health care but didn’t receive it (past year)	1.64 (1.06, 2.55)	**0.027**		
Feelings about family in general	1.77 (1.13, 2.78)	**0.014**		
Feelings about things you do with other people	2.23 (1.39, 3.59)	**0.001**	1.74 (1.01, 2.99)	**0.047**
**Need Factors**				
Multiple mental disorders (≥2)	1.74 (1.12, 2.70)	**0.014**		
Less severe cluster of mental disorder	2.18 (1.38, 3.44)	**0.001**		
Severe cluster of mental disorder	0.50 (0.31, 0.78)	**0.003**	0.59 (0.35, 0.97)	**0.039**
Substance dependence	1.80 (1.13, 2.86)	**0.013**		
Blood-borne Infectious diseases (HIV/HCV/HBV)	1.82 (1.16, 2.84)	**0.009**		
Multiple physical illness (≥2)	3.90 (1.75, 8.71)	**0.001**	2.74 (1.12, 6.70)	**0.027**
General Health (fair/poor)	2.12 (1.36, 3.31)	**0.001**	1.68 (1.01, 2.81)	**0.047**

*Adjusted odds ratios and confidence intervals are only shown for variables that remained significant in the final logistic regression model after backwards elimination.

Bolded p-values indicate significance at p < 0.05.

**Table 5:** **Unadjusted and adjusted odds ratios for associations between predictor variables and levels of service use for specialist health care visits (≥3 visits)**

Outcome variable	Unadjusted OR (95% CI)	P value	Adjusted OR (95% CI)*	P value
**Predisposing Factors**				
Age at enrolment visit				
Youth	0.33 (0.14, 0.78)	**0.011**		
25-44 years	0.44 (0.18, 1.05)	0.065		
> 44 years				
Education (≤Grade 8)	0.63 (0.37, 1.09)	0.097		
Hospitalized for mental illness (>2 times) in past 5 years	2.48 (1.35, 4.56)	**0.004**	2.53 (1.35, 4.75)	**0.004**
**Enabling Factors**				
Regular place to go for health care	2.32 (0.97, 5.57)	0.059		
Needed health care but didn’t receive it (past year)	0.66 (0.37, 1.17)	0.159		
Feelings about amount of time spent with other people	0.60 (0.31, 1.15)	0.122		
**Need Factors**				
Major Depressive Episode	1.71 (1.00, 2.94)	0.052	1.98 (1.11, 3.56)	**0.021**
Blood-borne Infectious diseases (HIV/HCV/HBV)	0.51 (0.26, 0.99)	**0.045**	0.48 (0.24, 0.97)	**0.042**

*Adjusted odds ratios and confidence intervals are only shown for variables that remained significant in the final logistic regression model.

Bolded p-values indicate significance at p < 0.05.

In the specialist care category (Table 5), having been hospitalized for a mental illness at least 2 or more times in the past 5 years and current major depressive episode were associated with high specialist service use, while having a blood-borne infectious disease was associated with lower odds of high specialist health service use. Age at enrolment was the only variable significant in univariate regression analyses at the p ≤ 0.05 level that was not present in the final regression model.

## Discussion

Contrary to our hypothesis, the application of the Gelberg-Anderson model within our sample of homeless mentally ill individuals revealed that those with greater assessed need, including severe mental disorders and blood-borne infectious diseases, accessed health services at significantly lower levels than those with lower assessed needs. The burden of illness in our sample was extremely high. More than half of participants met criteria for psychotic disorder, and over eighty-percent reported having multiple chronic physical illnesses. It was hypothesized that individuals with more severe mental disorders, multiple co-morbidities, and concurrent disorders, would have used health services at a higher frequency than those with less severe conditions. Further, based on findings from previous research using the Gelberg-Andersen model, it was expected that need-related factors would be strongly associated with higher levels of service use [15].

High health service use was defined as three or more visits in the past month, for both primary care and specialist visits. As such, 21% of participants accessed primary health services three or more times in the past month, while only 13% of participants accessed high levels of specialist health services. The vast majority of participants accessed primary or specialist services two or fewer times in the past month. This finding is consistent with other literature identifying that a small proportion of individuals tend to account for a disproportionately high amount of service use [23, 24]. While the 80^th^ percentile of the number of health services visits was chosen in order to define the outcome variable, it is important to note that even the median level of two visits in the past month is considerably greater than the number of health care visits per month that would be observed in the general population [25].

The frequency of service use was considered independently in the categories of primary care and specialist health service use for the purpose of differentiating between primary health services accessed by the individual (i.e., family physician, nurse, dentist, etc.), versus specialized referral-based health service use (i.e., specialist physician, psychiatrist, psychologist, etc.). In both categories, as expected, a greater number of need-related factors were significantly associated with level of service use than the other Gelberg-Andersen domains. Variables shown to be significantly associated with higher levels of health service use in previous studies such as substance use and female gender were non-significant in our models. It is possible that non-significant results observed for certain predictor variables could be due to small sample sizes within these cells. All individuals included in these analyses were recruited on the basis of current homelessness status and therefore it was not possible to show a relationship between homelessness and level of service use. However, previous studies using the Gelberg-Andersen framework have shown homelessness to be significantly associated with high service use compared to housed individuals, and thus these findings are understood in the context of higher average service use [15, 26].

### Primary health care visits

In the primary health care visit category, none of the predisposing factors were found to be significantly associated with level of health service use. Having a regular family physician, and negative feelings about ‘the things you do with other people’ were enabling factors associated with significantly greater odds of high service use. It is intuitive that participants who have regular family physicians would have higher levels of service use than those who do not have a regular family physician, as this is suggestive of health seeking behaviour. Feeling “horrible” about one’s social interactions may suggest a lack of positive social support and therefore an increased reliance on external sources, such as health services to meet needs.

Of the three need-related factors found to be significantly associated with level of service use, having multiple physical illnesses and reporting fair or poor general health were associated with higher levels of service use, supporting the hypothesis that people with poorer physical health ought to be accessing health services more frequently. Conversely, having a more severe mental disorder was associated with significantly lower likelihood of high health service use. This finding of lower health service use among those with more severe mental disorders (i.e. psychotic and bipolar disorder) is troubling and suggests possible gaps or barriers in the health system resulting in inadequate care for homeless individuals with more complex mental health challenges. The nature of such mental disorders can be such that individuals may not seek help when they need it due to stigma, mistrust in the medical system, negative past experiences, dissatisfaction with the prescription of medication without adequate psychological counseling and negative experiences with medication side-effects. This finding supports previous research that individuals experiencing homelessness and mental illness face barriers to service use [27, 28] and suggests that, in Vancouver, those with the most complex needs are particularly underserved.

### Specialist health care visits

The predisposing factor of hospitalization for a mental illness (>2 times) in the past 5 years was associated with higher levels of specialist health service use, suggesting that personal histories of specialized tertiary psychiatric care can help to explain increased levels of specialist care in the present. No enabling factors were significantly associated with specialist health service use. The only other factors associated with specialist health service use were need-related factors. Major depressive episode was associated with higher levels of specialist service use, suggesting that individuals with depression are likely to be referred to and make use of specialist services, including being seen by a psychiatrist or other mental health professional. Having a psychotic disorder, or more severe mental disorder, was not significantly associated with either high or low levels of specialist health care use. Given the difficulty in treating individuals with severe mental disorders and the limited availability of specialists, it is possible that this finding of non-significance may be related to the fact that such individuals are more likely to be turned away from specialist services or inadequately followed [29]. Finally, having a blood-borne infectious disease (i.e., HIV, HCV, or HBV) was associated with significantly lower specialist health service use, which may suggest that individuals with these conditions are underserved by specialist health care providers, or that these conditions can be successfully managed by primary health care providers.

### Strengths and limitations

The Gelberg-Andersen framework guided the selection of variables to be included in analyses and provided a useful means of organization into the three domains of predisposing, enabling and need-related factors. The variables available through the VAH study were defined in ways consistent with previous studies using the Gelberg-Andersen framework, and were relatively complete in scope to populate the three domains. Analyzing health service use within this framework enabled comparison between previously established findings that also used this framework and highlighted differences between our sample and those studied elsewhere. Our results represent the first application of the Gelberg-Andersen framework to a homeless mentally ill cohort in Canada.

Limitations include the fact that the data used were based on self-reported past-month service use and thus were subject to recall bias whereby individuals may have had difficulty accurately recalling the exact frequency and nature of all health services contacts. As well, participants may over or underreport certain types of service use due to social desirability bias or perceptions of stigma. Individuals experiencing homelessness and mental illness tend to be a ‘hard to reach’ and heterogeneous population and therefore it is difficult to generalize findings beyond our current sample. Further the cross-sectional design of this particular study does not allow us to make any direct causal inference about the association between level of need and service use. Efforts were made to ensure that as many established Gelberg-Andersen variables were included, however, certain variables might not have been included or may have been defined differently in comparison to previous studies. Additionally, inconsistencies between previous studies in the categorization of certain variables (i.e. substance use) within the three different Gelberg-Andersen domains, underscores the importance of judgment when placing particular variables into the three categories that comprise the model. While the overall sample size of the study allowed sufficient power to reduce the probability for a Type II error in the primary analysis, it is possible that the sample sizes for certain predictor variables (i.e. Aboriginal status) were not sufficiently large to establish a statistically significant.

## Conclusion

The current study found that homeless individuals with more severe mental disorders and blood borne infectious diseases had significantly lower odds of using high levels of primary and specialist health services respectively, despite evidence of need. Our results raise important questions concerning the adequacy of services available to homeless individuals who experience severe mental disorders. Insufficient involvement in community care may contribute to the further worsening of health and the high use of hospital services in this population. Strategies to better connect individuals experiencing homelessness with indicated services in the context of public, private and mixed models of health care delivery need to be developed to be responsive to individuals complex and unique needs.

## Abbreviations

AOR: Adjusted odds ratio
COPD: Chronic obstructive pulmonary disease
HBV: Hepatitis: B
HCV: Hepatitis C
HIV: Human immunodeficiency virus
RCT: Randomized control trial
VAH: Vancouver At Home.

## References

[1] Martens WH (2001). A review of physical and mental health in homeless persons. Public Health Rev.

[2] Fazel S, Khosla V, Doll H, Geddes J (2008). The prevalence of mental disorders among the homeless in western countries: systematic review and meta-regression analysis. PLoS Med.

[3] Hwang SW (2001). Homelessness and health. CMAJ.

[4] Frankish CJ, Hwang SW, Quantz D (2009). The relationship between homelessness and health: An overview of research in Canada. Finding Home: Policy Options for Addressing Homelessness in Canada edn.

[5] Hwang SW, Ueng JJM, Chiu S, Kiss A, Tolomiczenko G, Cowan L, Levinson W, Redelmeier DA (2010). Universal health insurance and health care access for homeless persons. Am J Public Health.

[6] Bonin J-P, Fournier L, Blais R, Perreault M, White ND (2010). Health and mental health care utilization by clients of resources for homeless persons in quebec city and montreal, Canada: a 5-year follow-up study. J Behav Health Serv Res.

[7] Bryant T, Leaver C, Dunn J (2009). Unmet healthcare need, gender, and health inequalities in Canada. Health Policy.

[8] Kushel MB, Perry S, Bangsberg D, Clark R, Moss AR (2002). Emergency department Use among the homeless and marginally housed: results from a community-based study. Am J Public Health.

[9] Kushel MB, Vittinghoff E, Haas JS (2001). Factors associated with the health care utilization of homeless persons. JAMA.

[10] Kuhn RR, Culhane DPD (1998). Applying cluster analysis to test a typology of homelessness by pattern of shelter utilization: results from the analysis of administrative data. Am J Community Psychol.

[11] Tsemberis SS, Gulcur LL, Nakae MM (2004). Housing First, consumer choice, and harm reduction for homeless individuals with a dual diagnosis. Am J Public Health (N Y).

[12] Stergiopoulos V, Dewa C, Durbin J, Chau N, Svoboda T (2010). Assessing the mental health service needs of the homeless: a level-of-care approach. J Health Care Poor Underserved.

[13] Stein JA, Andersen R, Gelberg L (2007). Applying the gelberg-andersen behavioral model for vulnerable populations to health services utilization in homeless women. J Health Psychol.

[14] Gelberg L, Andersen RM, Leake BD (2000). The Behavioral Model for Vulnerable Populations: application to medical care use and outcomes for homeless people. Health Serv Res.

[15] Lindamer LA, Liu L, Sommerfeld DH, Folsom DP, Hawthorne W, Garcia P, Aarons GA, Jeste DV (2011). Predisposing, enabling, and need factors associated with high service Use in a public mental health system. Adm Policy Ment Health.

[16] Stein JA, Andersen RM, Koegel P, Gelberg L (2000). Predicting health services utilization among homeless adults: a prospective analysis. J Health Care Poor Underserved.

[17] Bonin J-P, Fournier L, Blais R, Perreault M, White ND (2007). Are the responses of clients with psychiatric and addiction disorders using services for the homeless valid?. Can J Psychiatry.

[18] Somers JM, Patterson ML, Moniruzzaman A, Currie L, Rezansoff SN, Palepu A, Fryer K (2013). Vancouver At Home: pragmatic randomized trials investigating Housing First for homeless and mentally ill adults. Trials.

[19] Goering PN, Streiner DL, Adair C, Aubry T, Barker J, Distasio J, Hwang SW, Komaroff J, Latimer E, Somers J, Zabkiewicz DM (2011). The At Home/Chez Soi trial protocol: a pragmatic, multi-site, randomised controlled trial of a Housing First intervention for homeless individuals with mental illness in five Canadian cities. BMJ Open.

[20] Uttaro T, Lehman A (1999). Graded response modeling of the quality of life interview. Eval Program Plann.

[21] Sheehan DV, Lecrubier Y, Sheehan KH, Amorim P, Janavs J, Weiller E, Hergueta T, Baker R, Dunbar GC (1998). The Mini-International Neuropsychiatric Interview (MINI): the development and validation of a structured diagnostic psychiatric interview for DSM-IV and ICD-10. J Clin Psychiatry.

[22] Palepu A, Patterson M, Strehlau V, Moniruzzaman A, de Bibiana JT, Frankish J, Krausz M, Somers J (2013). Daily substance use and mental health symptoms among a cohort of homeless adults in Vancouver, British Columbia. J Urban Health.

[23] Chaput YJA, Lebel MJ (2007). Demographic and clinical profiles of patients Who make multiple visits to psychiatric emergency services. Psychiatr Serv.

[24] Dhingra S, Zack M, Strine T, Pearson W (2010). Determining prevalence and correlates of psychiatric treatment with Andersen’s behavioral model of health services use. Psychiatr Serv.

[25] Roos NP, Mustard CA (1997). Variation in health and health care Use by socioeconomic status in Winnipeg, Canada: does the system work well? Yes and No. Milbank Q.

[26] Pasic J (2005). High utilizers of psychiatric emergency services. Psychiatr Serv.

[27] Pauly B (2008). Harm reduction through a social justice lens. Int J Drug Policy.

[28] Whitehead M, Dahlgren G (2006). Concepts and principles for tackling social inequities in health: Levelling up Part 1. World Health Organization: Studies on Social and Economic Determinants of Population Health.

[29] Schulze B, Angermeyer MC (2003). Subjective experiences of stigma. A focus group study of schizophrenic patients, their relatives and mental health professionals. Soc Sci Med.

[CR30] The pre-publication history for this paper can be accessed here:http://www.biomedcentral.com/1472-6963/14/404/prepub

